# Lattice radiotherapy boost integrated with thoracic VMAT in bulky stage IIIB non–small cell lung cancer: a case report

**DOI:** 10.3389/fonc.2026.1965056

**Published:** 2026-10-28

**Authors:** Song Wang, Xing-Chuan Li, Jun-tao Ran, Quan-Lin Guan

**Affiliations:** 1 Department of Radiotherapy, The First Hospital of Lanzhou University, Lanzhou, China; 2 Department of Pediatrics, The Second Hospital of Lanzhou University, Lanzhou, China; 3 Department of General Surgery, The First Hospital of Lanzhou University, Lanzhou, China

**Keywords:** hypofractionated radiotherapy, immunotherapy, lattice radiotherapy, non-small cell lung cancer, spatially fractionated radiotherapy

## Abstract

**Background:**

Bulky unresectable stage III non-small cell lung cancer (NSCLC) presents a clinical challenge during definitive radiotherapy, as homogeneous dose escalation is often constrained by organ-at-risk (OAR) tolerance. Spatially fractionated radiotherapy (SFRT), including lattice radiotherapy (LRT), creates an intratumoral peak-valley dose distribution and may provide a means to intensify local dose delivery. However, the optimal dose, fractionation, vertex geometry, treatment sequence, and contribution of LRT to clinical response remain uncertain.

**Case presentation:**

We report a 41-year-old never-smoking Han Chinese man with bulky unresectable stage IIIB (cT4N2M0, AJCC 8th edition) disease, with a primary tumor measuring 71×53 mm at baseline. After two cycles of induction platinum-based chemotherapy, the disease was classified as stable disease. The patient subsequently received thoracic VMAT to 60 Gy in 15 fractions, during this course, a LRT plan was interleaved within the VMAT plan and delivered using an interleaved “4 VMAT treatments plus 1 LRT per week” pattern. Sixteen spheres were placed within the gross tumor volume (GTV), achieving a peak-to-valley dose ratio (PVDR) of 3.52. Serial CT showed a reduction in the sum of lesion diameters (SOD) of 20.7% after eight VMAT fractions, 29.6% after 14 fractions, and 40% at the 2-month post-treatment assessment. The patient developed grade 4 hematologic toxicity and grade 1 radiation pneumonitis after additional systemic therapy.

**Conclusion:**

This case demonstrates the delivery of weekly LRT during thoracic VMAT in a patient with bulky stage IIIB NSCLC, with significant tumor regression observed. This approach should be regarded as technically feasible in this case only, rather than as evidence that LRT can circumvent OAR (organs at risk) constraints or improve tumor control.

## 1 Introduction

For patients with unresectable stage III non-small cell lung cancer (NSCLC) who are suitable for combined-modality treatment, definitive concurrent chemoradiotherapy followed by consolidation immunotherapy remains a standard treatment strategy (1, 2). Nevertheless, patients with bulky primary tumors or extensive mediastinal disease remain at substantial risk of locoregional progression. Conventional homogeneous dose escalation has not consistently improved clinical outcomes and may increase severe treatment related toxicity (3). These limitations have stimulated interest in alternative approaches that intensify dose within the tumor while maintaining acceptable exposure of adjacent organs at risk (OARs).

Spatially fractionated radiotherapy (SFRT) deliberately delivers a heterogeneous dose distribution composed of high dose regions commonly termed “peaks” separated by lower dose regions (“valleys”) (4, 5). Lattice radiotherapy (LRT) is a three dimensional implementation of SFRT, typically delivered using intensity-modulated or volumetric-modulated arc therapy (IMRT/VMAT), in which spherical “vertices” are placed within the GTV to achieve focal dose intensification while respecting OAR constraints (6, 7). Unlike uniform dose escalation, LRT is intended to create spatially heterogeneous, focal intratumoral dose peaks rather than a homogeneous high dose across the whole tumor. Several biological mechanisms have been proposed to explain the potential effects of SFRT/LRT (6, 7). Published SFRT series have used widely varying peak doses, valley doses, vertex sizes, vertex spacing, peak-to-valley dose ratios (PVDR), and numbers of fractions (8). Comprehensive reporting of the spatial dose distribution including vertex geometry, valley dose, peak-to-valley metrics, dose gradients, and OAR exposure is essential (9, 10). Clinical experience with LRT/SFRT in bulky solid tumors is accumulating, yet the available evidence remains limited and consists largely of retrospective series, institutional experiences, and case reports (11, 16).

This report details a practical workflow in which a planned interleaved LRT boost was integrated into the thoracic VMAT radiotherapy for bulky stage IIIB squamous NSCLC. In the present case, the primary gross tumor volume (GTVp) measured 268.6cm³. Within the constraints of surrounding OARs, homogeneous dose escalation to such a large tumor is frequently infeasible; accordingly, the LRT boost (24 Gy in 3 fractions) selectively intensified the dose within the GTVp while preserving acceptable composite OAR dosimetry (bilateral lung V20 = 24.8%, esophageal Dmax=62.2 Gy). The principal aim of using LRT was to achieve focal dose escalation of the bulky residual primary tumor and, through intratumoral dose heterogeneity, to enhance local tumor shrinkage (6, 7, 12). We adopted an interleaved treatment strategy delivering the LRT once weekly during the VMAT course (60 Gy in 15 fractions) to first establish conformal PTV coverage and then selectively intensify the residual tumor, while avoiding the short-term concentrated high-dose pattern of a standalone lattice plan, thereby potentially minimizing cumulative OAR exposure during the lattice phase; this contrasts with the pre-VMAT sequencing approach reported by Zhou (11). The placement of the 16 spherical vertices in this study was guided by published thoracic LRT protocols (6, 7) and by the biological rationale of permitting immune engagement between fractions, as proposed by some studies (12, 13).

Given the heterogeneity of reported techniques, standardized reporting of LRT geometry, dose distribution, motion management, treatment delivery, and long-term toxicity is warranted to enable meaningful cross-study comparison and to define the role of LRT within multimodal stage III NSCLC treatment (9, 10).

## 2 Case presentation

A 41-year-old Han Chinese never-smoker presented with an intermittent cough persisting for one year, worsening over the preceding two months. He reported no dyspnea, hemoptysis, or weight loss. Physical examination revealed reduced breath sounds over the right upper lung field; Karnofsky performance status was 80. Contrast-enhanced chest CT (30 July 2025) demonstrated a bulky right upper-lobe mass (71×53mm) abutting the mediastinal pleura, with multiple enlarged mediastinal and right hilar lymph nodes (**Figures 1A, B**). Brain MRI and bone scan showed no distant metastasis. Bronchoscopic biopsy confirmed poorly differentiated squamous NSCLC (CK5/6 positive; TTF-1 and Napsin A negative). PD-L1 (22C3) tumor proportion score was <1%, and comprehensive driver-gene testing was negative. The clinical stage was cT4N2M0, stage IIIB (AJCC 8th edition). Tumor response was assessed on serial CT using RECIST v1.1 (sum of diameters, SOD) (19); toxicities were graded per CTCAE v5.0 (20).

**Figure 1:**
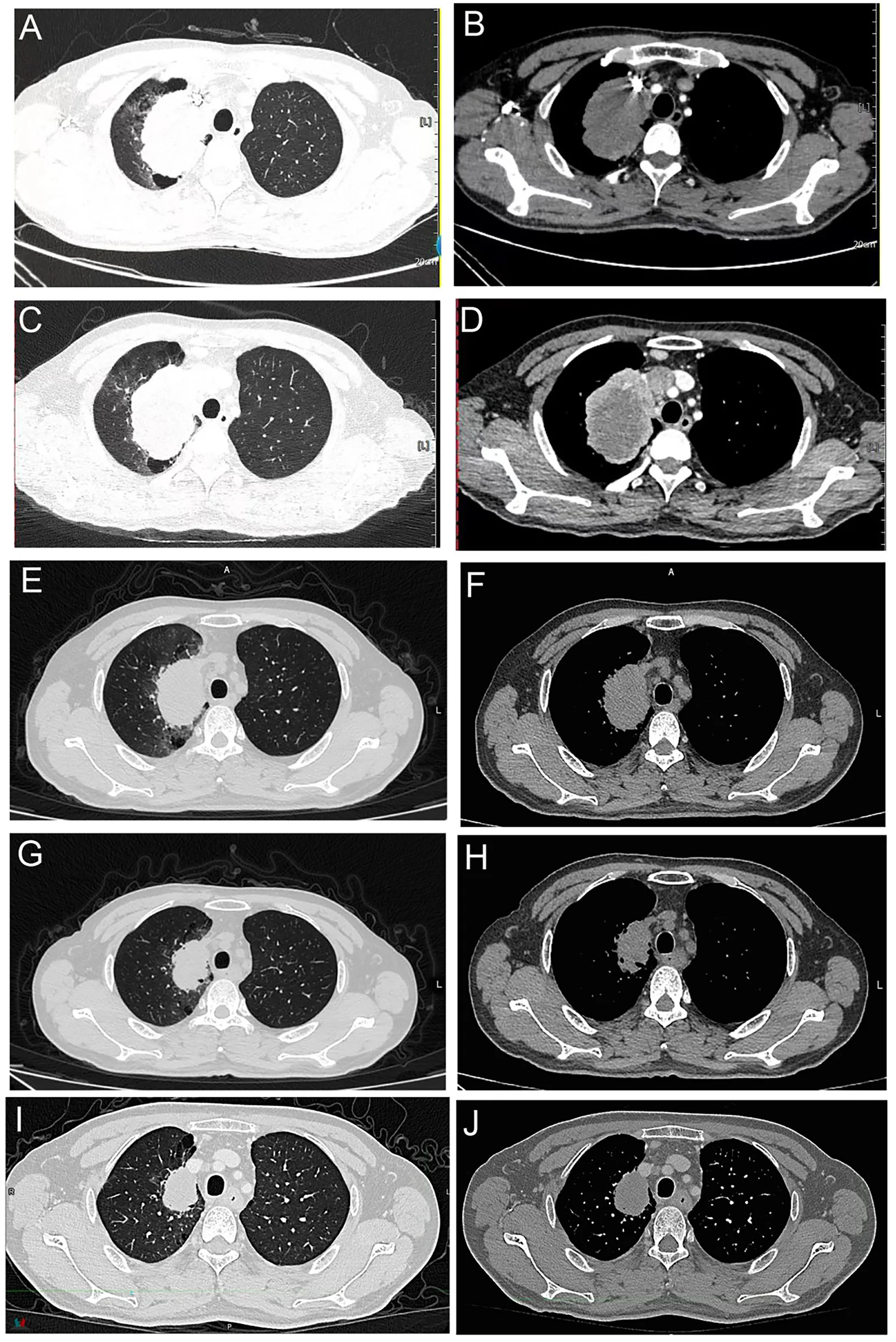
Serial chest CT demonstrating radiographic response during definitive hypofractionated chemoradiotherapy with a interleaved lattice boost in bulky unresectable stage IIIB squamous NSCLC. Paired axial CT images are shown in lung windows **(A, C, E, G, I)** and corresponding mediastinal windows **(B, D, F, H, J)** from baseline through follow-up, illustrating interval changes in the right upper-lobe primary tumor and mediastinal/right hilar nodal disease. **(A, B)** Baseline, 30 July 2025. **(C, D)** Post-induction chemotherapy restaging, 18 September 2025 (RECIST stable disease; SOD + 8.9%). **(E, F)** On-treatment assessment, 9 October 2025: early regression. **(G, H)** On-treatment reassessment, 21 October 2025: continued regression (time point prompting adaptive replanning). **(I, J)** Follow-up, 22 December 2025: sustained response without new thoracic progression. CT, computed tomography; NSCLC, non-small cell lung cancer; RECIST, Response Evaluation Criteria in Solid Tumors; SOD, sum of diameters.

The patient received two cycles of albumin-bound paclitaxel plus carboplatin on 5 August and 28 August 2025. Restaging CT on 18 September showed mild enlargement of the right pulmonary tumor (SOD + 8.9%) (**Figures 1C, D**). Given the substantial disease burden and lack of cytoreduction, the multidisciplinary team recommended treatment intensification. Chemoimmunotherapy was initiated on 19 September, combining albumin-bound paclitaxel, carboplatin, and tislelizumab.

Definitive thoracic radiotherapy commenced on 29 September. A 4D-CT simulation with contrast enhancement was performed; target volumes included the primary tumor (GTVp) and involved lymph nodes (GTVnd), with an internal target volume (ITV) generated by overlaying the GTV across all 10 respiratory phases. With the informed consent of the patient and his family, a LRT plan was added. The patient received 5 fractions (5F) of radiotherapy per week (including 4F VMAT and 1F LRT). VMAT delivered 60 Gy in 15F, 4F/week, prescribed to cover 95% of the PTV. LRT delivered 24 Gy in 3F, 1F/week.

During radiotherapy, the patient developed grade 2 myelosuppression with leukopenia, which improved with symptomatic and supportive care. Daily cone-beam CT (CBCT) image verification and registration were performed during radiotherapy. After eight fractions (4 VMAT + 1 LRT + 3 VMAT), the sum of diameters (SOD) had decreased by 20.7%; the radiotherapy target volumes were then modified and an adaptive plan was generated, and serial CT surveillance confirmed gradual tumor shrinkage. After 14 fractions (4 VMAT + 1 LRT + 4 VMAT + 1 LRT + 4 VMAT), the SOD had decreased by 29.6% (target lesion 56×39 mm). Follow-up CT scans (October 9 and October 21, 2025) showed ongoing tumor regression (**Figures 1E–H**). The patient completed the entire course of radiotherapy on October 22.

Additional chemoimmunotherapy was administered on October 24 and November 24, 2025, after which the patient developed grade 4 myelosuppression (nadir white blood cell count 1.42×10^9^/L; platelet count 21×10^9^/L), requiring growth factor support and platelet transfusion. At the 2-month assessment after radiotherapy completion (December 22, 2025), repeat CT showed a 40% reduction in SOD of the right pulmonary tumor (right-lung target lesion 43×38mm) (**Figures 1I, J**), fulfilling the partial response (PR) criteria of RECIST V1.1 (**Figures 2A, B**). Transient grade 1–2 gastrointestinal reactions and elevated liver function markers, as well as grade 1 radiation pneumonitis (ground-glass opacity within the irradiated field, asymptomatic), resolved with symptomatic medical management (**Figure 2C**); no other toxicities were observed. Starting December 23,2025, the patient received tislelizumab monotherapy as consolidation therapy every 21 days; the patient’s general condition was good and adverse effects were mild. Disease remained stable on imaging evaluations repeated every 2 months.

**Figure 2:**
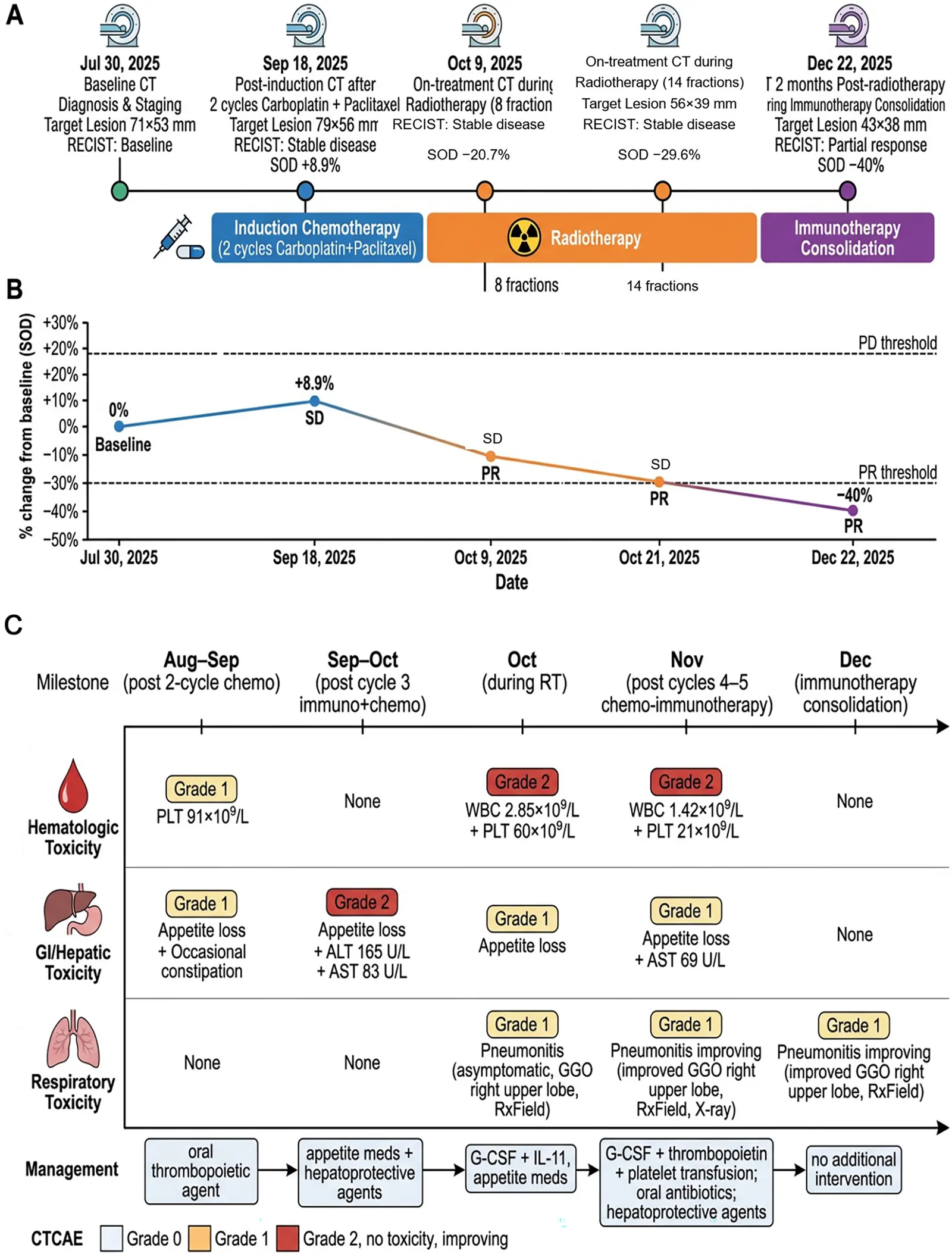
Treatment timeline, RECIST v1.1-based tumor response, and longitudinal summary of toxicities and management. **(A)** Timeline of treatment and imaging assessments with target-lesion measurements and RECIST classification (baseline 30 July 2025; restaging 18 September 2025: SD, SOD + 8.9%; on-treatment 9 October 2025: SD, SOD −20.7%; on-treatment 21 October 2025: SD, SOD −29.6%; follow-up 22 December 2025: sustained PR, SOD −40%). **(B)** Percentage change in SOD over time with RECIST thresholds for PR and PD. **(C)** Matrix-style timeline of treatment-related toxicities (CTCAE) and key supportive measures across treatment milestones, including predominantly hematologic toxicity, transient gastrointestinal/hepatic events, and field-limited radiation pneumonitis with subsequent improvement. CT, computed tomography; RECIST, Response Evaluation Criteria in Solid Tumors; SOD, sum of diameters; SD, stable disease; PR, partial response; PD, progressive disease; VMAT, volumetric modulated arc therapy; CTCAE, Common Terminology Criteria for Adverse Events; ALT, alanine aminotransferase; AST, aspartate aminotransferase; G-CSF, granulocyte colony-stimulating factor; GGO, ground-glass opacity.

### 2.1 Radiotherapy planning and lattice boost

Treatment machine. The medical linear accelerator used at our center was manufactured by the overseas manufacturer Elekta Limited (UK), model Elekta Infinity™; the domestic authorized agent for this device is Elekta (Shanghai) Medical Equipment Co, Ltd.

Treatment planning system. All treatment planning was performed using Monaco (Elekta Medical Systems, Sweden), version 5. The Monte Carlo dose algorithm was used for photon dose calculation. Dose was normalized to ensure that 95% of the PTV received the prescription dose. **Table 1** summarizes the LRT only and composite organ-at-risk (OAR) dosimetry. The composite dose distribution of the VMAT plan with the superimposed LRT boost is shown in **Figure 3**. The LRT boost was delivered as two non-coplanar VMAT arcs (couch 10° and 45°) on an Elekta Infinity™ linac, each covering a 230° gantry rotation with the collimator at 230° (130° equivalent) and traversing the 182°–52° interval in opposite directions to distribute the lattice vertices while sparing paraspinal organs at risk.

**Table 1:** Organ-at-risk (OAR) dosimetry for the lattice boost plan and the composite plan (definitive VMAT plus interleaved lattice boost).

OAR	Lattice boost plan			Composite plan		
	Dmax (Gy)	Dmean (Gy)	Dose-volume indices	Dmax (Gy)	Dmean (Gy)	Dose-volume indices
Bilateral lungs	15.3	0.9	V20 = 0.0%; V5 = 3.1%	80.6(including PTV)	14.6	V30 = 19.2%; V20 = 24.8%; V5 = 47.4%
Right lung	15.3	1.2	V20 = 0.0%; V5 = 6.0%	—	—	—
Heart	1.4	0.1	—	54.6	3.6	V40 = 0.6%; V30 = 1.5%; V20 = 2.9%
Esophagus	4.8	1.1	—	62.2	22.0	V50 = 19.1%; V40 = 33.7%; V30 = 39.5%
Spinal cord	5.1	0.8	—	33.9	11.7	V30 = 3.4%

Dmax, maximum dose; Dmean, mean dose; Vx, percentagevolume receiving ≥ x Gy; “—” indicates not applicable or not reported. The composite plan represents the cumulative dosimetry from definitive VMAT (60 Gy in 15 fractions) and the interleaved lattice boost (24 Gy in 3 fractions). Patient positioning and target localization were verified using daily cone-beam CT (CBCT).

**Figure 3:**
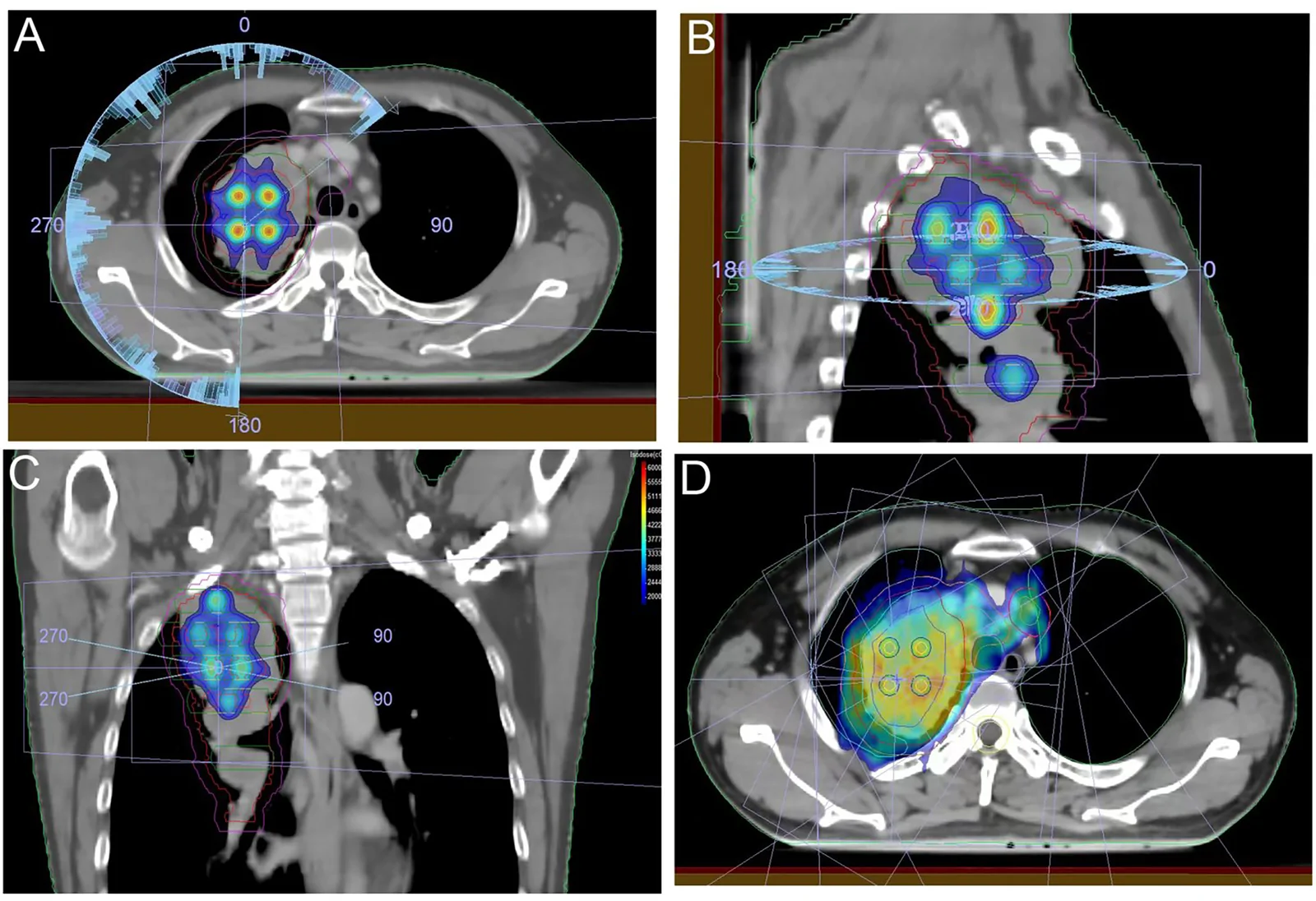
Composite dose distribution of definitive VMAT with a interleaved lattice radiotherapy (LRT) boost for bulky stage IIIB NSCLC. Dose color wash and isodose lines on CT simulation images demonstrate focal intratumoral dose intensification (“peaks”) with surrounding lower-dose regions (“valleys”) and dose fall-off to adjacent organs at risk. **(A)** Axial view. **(B)** Sagittal view. **(C)** Coronal view. **(D)** Additional axial level highlighting intratumoral peak-valley heterogeneity. CT, computed tomography; VMAT, volumetric modulated arc therapy; LRT, lattice radiotherapy; NSCLC, non-small cell lung cancer.

VMAT plan. The prescription dose was 60 Gy in 15F (EQD2: 70Gy, BED10: 84 Gy). Definitive radiotherapy was delivered using an Elekta Infinity™ linear accelerator with a 6-MV X-ray beam. Eight partial arcs were used, with the couch at 0° (coplanar). The total monitor units (MU) for the VMAT plan were approximately 2,107 MU.

Lattice boost plan. The lattice boost was delivered on an Elekta Infinity™ linac using two non-coplanar 6-MV FFF VMAT arcs (couch 10° and 45°), planned in Monaco with the Monte Carlo algorithm. Both arcs shared a single isocenter, covered a 230° gantry rotation with the collimator at 230° (130° equivalent) traversing the 182°–52° interval in opposite directions, and delivered 2507 MU and 3171 MU per fraction, respectively (5678 MU/fraction total). Patient-specific QA for the LRT plan used the same diode array with the same gamma criteria. Pre-treatment cone-beam CT (CBCT) was performed before each fraction to verify vertex positioning relative to the GTVp (primary gross tumor volume), and offline review was performed to assess intrafraction motion.

Patient-specific QA: Patient-specific quality assurance (QA) was performed using an ArcCHECK diode-array phantom. Gamma analysis of both the VMAT and LRT plans employed a 3%/2 mm dose-difference/distance-to-agreement criterion, a 10% low-dose threshold, and global normalization, yielding pass rates of 98.6% (VMAT) and 93.7% (LRT). Given the detector sampling characteristics of the ArcCHECK device, the LRT plan was re-evaluated using a 3%/3 mm dose-difference/distance-to-agreement criterion, which gave a pass rate of 95.2%; under this criterion, the pass rates of both plans exceeded 95%.

Vertex geometry. In the LRT plan, 16 spheres were placed within the GTVp (volume 268.6cm³), with a total sphere volume of 6 cm³(each sphere 0.89 cm in diameter; center-to-center spacing between adjacent spheres 1.76 cm). Sphere placement was performed using three-dimensional model construction, covering the radiologically densest and most metabolically active regions of the tumor based on clinical judgment, while sparing a 1 cm margin from the upper-lobe bronchus and maintaining 0.5 cm from the tumor boundary. The spheres were arranged in a hexagonal (honeycomb) pattern across three axial planes (the upper, middle, and lower thirds of the GTVp); arrangement feasibility was confirmed by point-arrangement verification and was consistent with published practice (17, 18). The volume ratio of the spheres to the GTVp was 2%. The sphere-localization model was generated by a medical physicist at our institution, which may introduce inter-operator variability.

Within the LRT plan, the prescription dose was 24 Gy/3F(EQD2: 36Gy, BED10: 43.2 Gy). The hotspot physical dose of the spheres (peak dose) was Dmax 35.54 Gy, and the mean physical dose of the spheres was Dmean 27.66 Gy; the sphere D10 was 30.85 Gy, D90 was 24.93 Gy, and D95 was 24.44 Gy. The Dmean(95-100%) of the Lattice Volume (VL) was 1.14 Gy. The mean dose to the gross tumor volume (GTVp excluding the spheres, the valley dose) was 10.09 Gy. According to Wu et al. (2020) (6), the VPDR (valley-to-peak dose ratio) is defined as Dmean(95-100%) of the Lattice Volume (VL) divided by the prescription peak dose; thus the VPDR was 1.14/24 = 0.048, and the PVDR (peak-to-valley dose ratio) was 21.05. Following Reiff et al.’s definition (21) of PVDR, physical sphere dose Dmax divided by the valley dose, the PVDR was 35.54/10.09 = 3.52. Following Yang et al.’s definition (22), PVDR-mean was 2.74 (peak Dmean/valley Dmean, 27.66/10.09). Other algorithms have also been reported in some studies, and the calculation of PVDR has not yet been unified.

Adaptive replanning and dose accumulation. On-treatment CT imaging was acquired on October 9 and October 21, 2025. Owing to the observed tumor shrinkage, both the VMAT and LRT plans were replanned using the CT dataset obtained on Follow-up CT: the primary GTVp, involved nodal GTV, ITV, and PTV were modified, and the lattice vertices were repositioned according to the updated GTV. In the new adaptive plan, 7 spheres were placed within the GTVp (volume 103.8 cm³), with a total sphere volume of 2.46 cm³ (each sphere approximately 0.89 cm in diameter; center-to-center spacing between adjacent spheres 1.76 cm). For composite dosimetry, the individual dose distributions were accumulated based on the two CT datasets; plan merging was performed using the Monaco system, with registration achieved by rigid image registration, registration quality assessed via anatomic landmarks, and the accumulated dose calculated using a dose-warping method. After superimposing the VMAT and LRT plans, the organs at risk—both lungs, right lung, heart, and others—were all within the dose constraints (see **Table 1**), which also lists the OAR constraints for the LRT plan alone.

Rationale for treatment modifications. This patient had a large pulmonary mass. Following induction chemotherapy, the tumor increased in size, suggesting that the initial systemic therapy was suboptimal. We therefore intensified local therapy with a VMAT-plus-LRT plan to maximize tumor shrinkage; serial CT during radiotherapy confirmed tumor regression, supporting the efficacy of this intensified local approach.

### 2.2 Follow-up and outcomes

Clinician-assessed outcomes. Serial imaging was performed at baseline and at each specified timepoint, with treatment response evaluated using RECIST v1.1. The sum of diameters (SOD) was 71×53 mm at baseline; after 8 fractions the SOD had decreased by 20.7%, by 29.6% after 14 fractions (target lesion 56×39 mm), and by 40% at the 2-month follow-up (target lesion 43×38 mm; 22 December 2025), fulfilling criteria for partial response.

Patient-assessed outcomes. The patient’s Karnofsky performance status (KPS) was 80 at presentation and remained stable through treatment; symptomatic improvement (reduced cough) was reported during treatment. The patient’s KPS was 80 at the 2-month follow up after radiotherapy.

Diagnostic follow-up. Contrast-enhanced CT demonstrated progressive tumor shrinkage; comprehensive follow-up imaging showed no tumor progression.

Intervention adherence and tolerability. The patient completed all prescribed radiotherapy (100% adherence). After radiotherapy, two additional cycles of chemotherapy were administered; following the occurrence of grade 4 myelosuppression, chemotherapy was subsequently paused, and tislelizumab monotherapy was given as consolidation therapy every 21 days.

Adverse events. All adverse events were graded according to CTCAE v5.0 and are summarized in **Figure 2C**. Two months after radiotherapy, ground-glass opacity was observed within the irradiated field, the patient was asymptomatic (no cough or expectoration), and this was considered grade 1 radiation pneumonitis, which improved with symptomatic treatment and resolved spontaneously without corticosteroids, with the ground-glass opacity disappearing. After radiotherapy, the patient received two additional cycles of chemotherapy; following the occurrence of grade 4 myelosuppression (WBC nadir 1.42×10^9^/L; platelets 21×10^9^/L), the patient improved with G-CSF support and platelet transfusion, and chemotherapy was subsequently paused. The patient was otherwise well.

## 3 Discussion

LRT can generate focal intratumoral dose peaks; very-high-dose peaks may induce direct tumor cell kill, endothelial injury, vascular perturbation, and local changes in oxygenation and stromal signaling. Lower-dose valleys may preserve partially injured but viable tumor and stromal compartments, potentially facilitating bystander or abscopal-type effects through cytokine signaling, extracellular vesicles, immune cell recruitment, and modulation of antigen presentation. Preclinical and translational studies further suggest that spatial dose heterogeneity may promote immunogenic cell death, dendritic cell activation, and T cell priming (12, 13). However, these mechanisms remain incompletely characterized, and the relative contributions of peak-dose effects, valley-dose effects, vascular injury, and immune modulation in patients have not yet been established. Notably, the optimal dose and fractionation for an immunologic effect remain uncertain. Higher peak doses may enhance direct tumor injury but can also increase necrosis, bleeding, edema, or damage to adjacent normal tissues; conversely, lower valley doses may spare normal tissue but may be insufficient for tumor control in the inter-vertex regions. Therefore, the biological interpretation of LRT should not rest on peak dose alone, and comprehensive reporting of the spatial dose distribution including vertex geometry, valley dose, peak-to-valley metrics, dose gradients, and OAR exposure is essential. Both the LRT plan and sphere placement require further investigation and exploration. Nevertheless, in patients with bulky tumors, the interleaving of lattice radiotherapy within VMAT should be regarded as an observation of technical and clinical feasibility rather than as evidence that LRT can circumvent OAR constraints or improve tumor control.

Because LRT and VMAT are interleaved within the same course, the tumor shrinkage observed during treatment provides a basis for adaptive replanning of subsequent fractions (including vertex repositioning), making this interleaved pattern attractive from an operational standpoint, and it was technically feasible in this case. That said, it may also introduce important uncertainties: tumor regression renders the vertex geometry and dose distribution dependent on adaptive planning, and the local response cannot be attributed solely to the LRT boost. In this patient, measurable tumor shrinkage was already observed after fractions 8 and 14 (which had incorporated the delivered LRT boost), that is, before completion of the entire radiotherapy course; in addition, chemotherapy and immunotherapy were administered concurrently throughout the treatment period. Therefore, tumor shrinkage cannot be specifically attributed to LRT. The observed response likely reflects the combined effect of hypofractionated VMAT, the superimposed LRT boost, systemic therapy, and the shrinkage occurring during treatment.

No biomarkers, immune cell analyses, or prospective controlled data are available to establish an LRT related immune effect; however, it is worth noting that the deliberately created intratumoral high-low dose heterogeneity produced by LRT may exert a synergistic, potentiating effect with subsequent PD-1-blocking immunotherapy represented by tislelizumab, a hypothesis that remains to be validated in larger-scale studies (14, 15).

## 4 Conclusion

This case demonstrates the clinical implementation in a patient with bulky stage IIIB NSCLC, who received thoracic VMAT radiotherapy using an interleaved “4 VMAT treatments plus 1 LRT per week” pattern, with the LRT plan interleaved among the VMAT plans. The treatment employed a three-dimensional vertex-based dose distribution and was delivered concurrently with systemic therapy. Clinical responses appeared after radiotherapy fractions 8 and 14, and the effects of chemotherapy, immunotherapy, VMAT, adaptive replanning, and LRT cannot be precisely distinguished. Therefore, this report should be interpreted as a technical feasibility observation from short-term clinical follow-up rather than as evidence of LRT efficacy or immune synergy. Future studies should adopt standardized reporting, including vertex geometry, motion management, dose calculation, patient-specific quality assurance (QA), organ-at-risk (OAR) constraints, biological dose, dose accumulation, and long-term toxicity.

## Data Availability

The original contributions presented in the study are included in the article/supplementary material. Further inquiries can be directed to the corresponding author.
